# Supplementary material for: A prefrontal motor circuit initiates persistent movement
Source: Nat Commun. 2024 Jun 19;15:5264. doi: 10.1038/s41467-024-49615-0 (PMC11187183; doi:10.1038/s41467-024-49615-0)
Supplement: Supplementary file 3 — Description of Additional Supplementary Files [file 41467_2024_49615_MOESM3_ESM.pdf]

File name: Supplementary Movie 1
Description: Non-persistent movement.

File name: Supplementary Movie 2
Description: Persistent movement.
