# Supplementary material for: A prefrontal motor circuit initiates persistent movement
Source: Nat Commun. 2024 Jun 19;15:5264. doi: 10.1038/s41467-024-49615-0 (PMC11187183; doi:10.1038/s41467-024-49615-0)
Supplement: Supplementary file 6 — Reporting Summary [file 41467_2024_49615_MOESM6_ESM.pdf]

nature portfolio

Corresponding author(s): Qian-Quan Sun

Last updated by author(s): Mar 28, 2024

Reporting Summary

Nature Portfolio wishes to improve the reproducibility of the work that we publish. This form provides structure for consistency and transparency in reporting. For further information on Nature Portfolio policies, see our Editorial Policies and the Editorial Policy Checklist.

Statistics

For all statistical analyses, confirm that the following items are present in the figure legend, table legend, main text, or Methods section.

n/a	Confirmed	
☐	☒	The exact sample size (*n*) for each experimental group/condition, given as a discrete number and unit of measurement
☐	☒	A statement on whether measurements were taken from distinct samples or whether the same sample was measured repeatedly
☐	☒	The statistical test(s) used AND whether they are one- or two-sided<br>*Only common tests should be described solely by name; describe more complex techniques in the Methods section.*
☒	☐	A description of all covariates tested
☐	☒	A description of any assumptions or corrections, such as tests of normality and adjustment for multiple comparisons
☐	☒	A full description of the statistical parameters including central tendency (e.g. means) or other basic estimates (e.g. regression coefficient) AND variation (e.g. standard deviation) or associated estimates of uncertainty (e.g. confidence intervals)
☐	☒	For null hypothesis testing, the test statistic (e.g. *F*, *t*, *r*) with confidence intervals, effect sizes, degrees of freedom and *P* value noted<br>*Give P values as exact values whenever suitable.*
☒	☐	For Bayesian analysis, information on the choice of priors and Markov chain Monte Carlo settings
☒	☐	For hierarchical and complex designs, identification of the appropriate level for tests and full reporting of outcomes
☐	☒	Estimates of effect sizes (e.g. Cohen's *d*, Pearson's *r*), indicating how they were calculated

Our web collection on statistics for biologists contains articles on many of the points above.

Software and code

Policy information about availability of computer code

Data collection	Neural activity and behavioral were collected using RHX Data Acquisition Software, available from https://intantech.com/RHX_software.html
Data analysis	Connectivity estimation used TSPE toolbox (https://github.com/biomemsLAB/TSPE)<br>Estimated firing rate using Bayesian adaptive kernel smoother (https://github.com/nurahmadi/BAKS)<br>Identified opto-tag single-units using stimulus-associated spike latency test (http://kepecslab.cshl.edu/software/)<br>Spike sorting procedures were performed with an offline software Spikesorter (http://swindale.ecc.ubc.ca/home-page/software/)<br>Other codes are freely available from https://doi.org/10.5281/zendo.7953792.

For manuscripts utilizing custom algorithms or software that are central to the research but not yet described in published literature, software must be made available to editors and reviewers. We strongly encourage code deposition in a community repository (e.g. GitHub). See the Nature Portfolio guidelines for submitting code & software for further information.

Data

Policy information about availability of data

All manuscripts must include a data availability statement. This statement should provide the following information, where applicable:

- Accession codes, unique identifiers, or web links for publicly available datasets
- A description of any restrictions on data availability
- For clinical datasets or third party data, please ensure that the statement adheres to our policy

Source data are provided with this paper. Raw data are not currently available through public database, but they can be requested from authors

Research involving human participants, their data, or biological material

Policy information about studies with human participants or human data. See also policy information about sex, gender (identity/presentation), and sexual orientation and race, ethnicity and racism.

Reporting on sex and gender	N/A
Reporting on race, ethnicity, or other socially relevant groupings	N/A
Population characteristics	N/A
Recruitment	N/A
Ethics oversight	N/A

Note that full information on the approval of the study protocol must also be provided in the manuscript.

Field-specific reporting

Please select the one below that is the best fit for your research. If you are not sure, read the appropriate sections before making your selection.

☒ Life sciences ☐ Behavioural & social sciences ☐ Ecological, evolutionary & environmental sciences

For a reference copy of the document with all sections, see nature.com/documents/nr-reporting-summary-flat.pdf

Life sciences study design

All studies must disclose on these points even when the disclosure is negative.

Sample size	For chemo-genetic experiments, 4 mice were used. For optogenetic experiments, 4 mice were used. For electrophysiological recording, 6 mice were used. We used more than 3 mice to ensure an adequate statistical power and exclude individual difference.
Data exclusions	For tail shocking task, the mice who did not perform escaping behavior were excluded from the test.
Replication	Major findings are repeated in different mice using different approaches, such as optogentics and chemo-genetics.
Randomization	No comparison was performed between different groups of mice. All experimental comparison was performed within group, such as w/o laser. Laser was randomly delivered to mice.
Blinding	Data collection and analysis were not blinded to the investigator. However, all data analysis were conducted through semi-automatic codes. These codes are designed prior to the analysis. It is equivalent to the blinding analysis.

Reporting for specific materials, systems and methods

We require information from authors about some types of materials, experimental systems and methods used in many studies. Here, indicate whether each material, system or method listed is relevant to your study. If you are not sure if a list item applies to your research, read the appropriate section before selecting a response.

Materials & experimental systems

n/a	Involved in the study	
☒	☐	Antibodies
☒	☐	Eukaryotic cell lines
☒	☐	Palaeontology and archaeology
☐	☒	Animals and other organisms
☒	☐	Clinical data
☒	☐	Dual use research of concern
☒	☐	Plants

Methods

n/a	Involved in the study	
☒	☐	ChIP-seq
☒	☐	Flow cytometry
☒	☐	MRI-based neuroimaging

Animals and other research organisms

Policy information about studies involving animals; ARRIVE guidelines recommended for reporting animal research, and Sex and Gender in Research

Laboratory animals	All experimental mice were bred on a C57/BL6J background and housed at 21-23 Celsius with 25%-30% humidity. For viral injection, P14-30 mice were used. For behavioral test, 2-5 month old mice were used.
Wild animals	No wild animal is used in this study
Reporting on sex	Male and female mice were compared on behavioral task
Field-collected samples	No field-collected samples
Ethics oversight	All experimental procedures were approved by the Institutional Animal Care and Use Committee (IACUC) and the Biosafety Committee of the University of Wyoming

Note that full information on the approval of the study protocol must also be provided in the manuscript.
